# Review of cognitive behavioural therapy mobile apps using a reference architecture embedded in the patient-provider relationship

**DOI:** 10.1186/s12938-018-0611-4

**Published:** 2018-12-17

**Authors:** Alice Lan, Alexandra Lee, Kristin Munroe, Cameron McRae, Linda Kaleis, Karim Keshavjee, Aziz Guergachi

**Affiliations:** 1 University of Toronto, Health Sciences Bldg, 155 College St, 4th Floor, Toronto, ON M5T 1P8 Canada; 2 Ryerson University, 111 Gerrard Street East, Unit 301, Toronto, ON M5B 1G8 Canada; 3 Ryerson University, 350 Victoria Street, Toronto, ON M5B 2K3 Canada

**Keywords:** mHealth, Mobile app, Cognitive behavioural therapy, Mental health, Virtual care

## Abstract

**Background:**

Mobile health apps (mHealth apps) are increasing in popularity and utility for the management of many chronic diseases. Although the current reimbursement structure for mHealth apps is lagging behind the rapidly improving functionality, more clinicians will begin to recommend these apps as they prove their clinical worth. Payors such as the government or private insurance companies will start to reimburse for the use of these technologies, especially if they add value to patients by providing timely support, a more streamlined patient experience, and greater patient convenience. Payors are likely to see benefits for providers, as these apps could help increase productivity between in-office encounters without having to resort to expensive in-person visits when patients are having trouble managing their disease.

**Key findings:**

To guide and perhaps speed up adoption of mHealth apps by patients and providers, analysis and evaluation of existing apps needs to be carried out and more feedback must be provided to app developers. In this paper, an evaluation of 35 mHealth apps claiming to provide cognitive behavioural therapy was conducted to assess the quality of the patient-provider relationship and evidence-based practices embedded in these apps. The mean score across the apps was 4.9 out of 20 functional criteria all of which were identified as important to the patient-provider relationship. The median score was 5 out of these 20 functional criteria.

**Conclusion:**

Overall, the apps reviewed were mostly stand-alone apps that do not enhance the patient-provider relationship, improve patient accountability or help providers support patients more effectively between visits. Large improvements in patient experience and provider productivity can be made through enhanced integration of mHealth apps into the healthcare system.

## Background

Cognitive behavioral therapy (CBT) is an evidence-based therapeutic approach used to treat psychological distress and a variety of mental disorders [1]. This therapy aims to modify maladaptive cognitions that lead to distress and problematic behaviours, thereby reducing negative symptoms and improving functioning [2]. CBT has been shown to produce large effect size improvements for treatment of mental health disorders, such as anxiety and depression [3]. CBT can be paired with pharmaceutical treatments to improve outcomes and has proven to be more effective than antidepressants when used for the treatment of depression in adults [3].

One method of CBT delivery that has been proven effective is internet-based CBT (iCBT), which has led to symptom reduction in both small and large effect sizes [4]. In this treatment method, a licensed therapist supports patients through online messaging platforms, e-mail, or web pages and provides them with exercises and behavioural intervention programs [5, 6]. iCBT has been identified as a plausible alternative to traditional CBT for patients with depression; helping to improve patient outcomes [7]. Randomized controlled trials have also shown that therapist-assisted iCBT is comparable to face-to-face CBT [8, 9], even when considering regarding development of strong patient-provider relationship [10].

With this shift towards alternative delivery methods for mental health therapies, an increasing number of mobile health (mHealth) apps in the mobile marketplace have emerged claiming to provide CBT. In contrast with iCBT, mHealth CBT apps tend to be self-guided and it is unknown if these apps effectively implement the evidence-based principles of CBT [11–14]. Additionally, there is little evidence demonstrating that these CBT apps can be recommended for unsupervised self-management [15]. The small existing evidence base is further exacerbated by the fast pace of technology relative to the pace of research and evaluation of mHealth apps [16]. Further research is required to better understand the mHealth CBT apps marketplace, particularly related to the effect on patient-provider relationships [17]. In addition, while research demonstrates patient interest in using mHealth apps for self-management, clinician interaction and health system integration of app has been identified as an important factor for patient confidence and ultimate behaviour change [18].

The purpose of this paper is to apply an mHealth app evaluation framework to CBT mHealth apps, to better understand the current marketplace for CBT mHealth apps, focusing primarily on the presence of functionalities to support patient-provider relationships. Specifically, this paper will focus on apps targeted towards adults with depression and/or anxiety.

## Framework development

An evaluation framework was developed to evaluate the quality of the patient-provider relationship in CBT mHealth apps based on a reference architecture for health app design [19], (see Table 1). The evaluation framework is composed of 20 measures aimed at measuring the evidence-based support of CBT mHealth apps and their ability to enhance the patient-provider relationship. These 20 measures were based on properties from Chindalo et al. reference architecture which distinguishes features such as explicitly identifying patient’s diagnosis, enabling interoperability with EMRs, identifying and tracking process and proxy metrics for diseases as well as identifying and tracking important outcome measures [19]. These concepts fit with Albrecht et al. framework which provides details on evidence-based criteria that should be considered when evaluating mobile applications [20]. The framework also identifies features that are based on the patient engagement framework created by Balouchi et al. which focuses on functionalities of mobile apps that enhance the patient-provider relationship [21]. The rationale for the methodology is to provide a perspective on the experience of general users and clinicians when identifying mHealth apps for the purpose of CBT.

**Table 1:** Ranking of functionalities

Function	Number of apps with function
Physiological measurement	0
Lab results	0
Medications	0
Integrations	0
Health system utilization	2
Predictive analytics	3
Prescribed	3
Patient information	4
Patient reported outcomes and experience measures (PREMS/PROMS)	4
Safety issues	4
Patient risk behaviours (e.g., smoking)	5
Community resources	7
Social supports	8
Diagnosis	12
Notifications	14
Incentives to use	18
Symptoms	18
Education and recommendations	22
User interface	24
Behaviour tracking (e.g., using sensors)	24

The final list of measures was developed with an experienced clinician (KK) and took into account the information needed to provide high quality clinical care to a patient requiring CBT. The measures developed were customized for the treatment of mental health disorders, such as depression and anxiety; diseases that respond to CBT. Although some of the measures can be used for evaluating other disease types, the set of measures developed for CBT are only appropriate for mental health and related disorders.

## Methods

50 CBT mHealth apps were identified from the Apple iTunes and Google Play app stores using the search terms “Cognitive Behavioural Therapy” or “CBT.” The rationale for the use of the health app design reference architecture over other popular frameworks used for mHealth App reviews is described previously [19].

Each app was downloaded and screened independently against 20 functional measures by two reviewers. Each measure was scored on a binary scale (0,1). Apps received a score of 1 if they had at least one attribute of that measure. To generate an evaluation score for each app, the sum of the binary measures was taken. The agreement between scores was determined after a blind independent review. The agreement between scores was completed by examining the number of scores the reviewers agreed on divided by the total number of functions in the framework. The mean evaluation score was calculated and used for analysis.

Before screening initiation, a calibration exercise was carried out with five randomly selected mHealth apps, which were evaluated by six reviewers. The calibration allowed the areas of discrepancies in interpretation of measures to be surfaced and addressed and improved the standardization of the approach. All reviewers were trained in the standardized method, and each of the 50 apps was evaluated by two independent reviewers.

Reviewers gave their ratings and included descriptions justifying their decision for each measure. After evaluations were complete, all data were collated into a single spreadsheet. Before data analysis, 15 apps identified by reviewers were excluded as they did not claim to provide CBT and offered other functions unrelated to the patient-provider relationship. Reviewers downloaded the app and scored them using the standardized method. Each app was independently and blindly screened against the evaluation criteria. For each of the measures, the higher score between the two reviewers was accepted, and final scores were generated for each app. The complete list of apps downloaded can be found in Appendix 1.

## Results

**Fig. 1:**
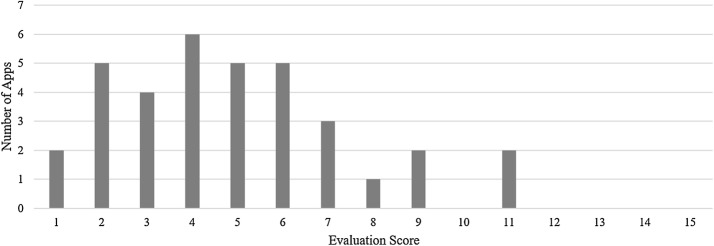
Distribution of app evaluation scores

The mean evaluation score across the 35 apps was 4.9 out of 20 functional criteria. The median score was 5. The two highest apps met 11 out of 20 functional criteria. The lowest app met 2 out of 20 functional criteria (see Fig. 1).

Overall, the apps scored well on functions including education and recommendations, user interface, and behaviour tracking functional criteria (See Table 1). Primarily, these criteria were met through the provision of education about CBT techniques and how they may reduce patient symptoms. Apps generally scored poorly on criteria including physiological measurement, patient health information collection, lab results, medication or comorbidities as well as health system integration and utilization; all of which may be important for management of patient with mental health disorders.

## Discussion

**Fig. 2:**
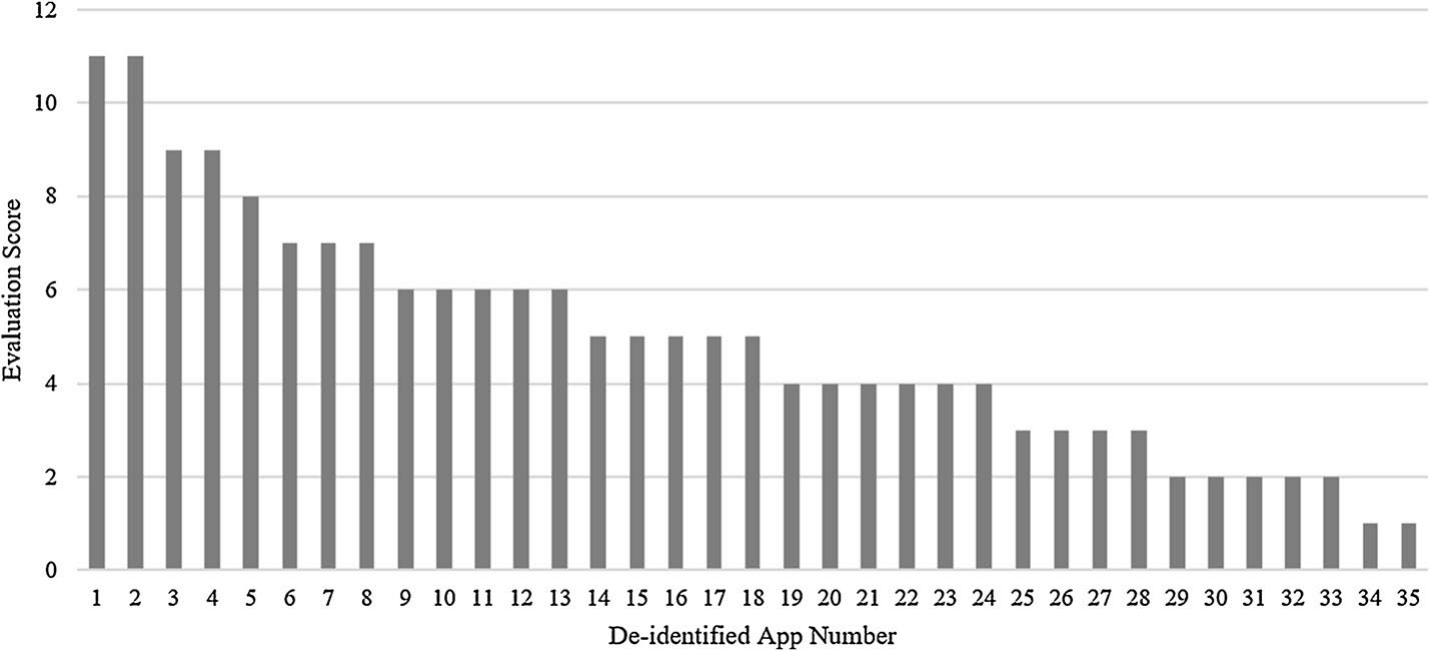
App evaluation scores after download

While recent literature suggests the potential of mHealth apps to improve care accessibility and decrease depression levels in users, findings from this research suggest that the current marketplace for mHealth apps is limited in its ability to provide benefits for the patient-provider relationship [12, 13]. Overall, our research found that the mHealth apps in the marketplace primarily act only as symptom trackers or educational resources with little integration into the larger healthcare system (see Fig. 2).

While apps overall did not score high on the evaluation framework, particularly in regards to healthcare integration, it should be noted that apps that perform only one core function may still provide some benefit to users. For instance, one empirical study reported the use of CBT-based depression apps are especially useful when they provide mood prediction; demonstrating the potential benefits of apps containing this feature alone [22]. Since our criteria were used to evaluate overall prevalence of functions as well as market gaps and opportunities, effectiveness of the individual functions was not taken into consideration.

Overall, by not providing healthcare integration, the apps under review did not provide opportunities for ensuring patient accountability and presented very little opportunity for use by healthcare providers. In addition, this lack of integration with providers and the healthcare system as a whole may limit the effectiveness of these apps in supporting sustained behaviour change [18]. It has been argued that mHealth apps should not be designed for healthcare provider use, and instead their main purpose is for patient empowerment outside the provider-patient relationship, suggesting their utility despite lack of integration. For instance, recent studies have found that mHealth apps may be useful and effective when used for self-monitoring and providing support for patients who are interested in self-treatment [23]. Therefore, apps that scored low on our evaluation criteria may present utility for highly motivated patients who are self-starters. Additional areas of improvement identified for the apps include more meaningful use of data collected, a stronger evidence base, and the ability to send notifications.

Identified limitations of the study are as follows: (1) the research team was not able to establish how often apps were used, or by which populations; (2) no patient representatives were included in the creation of the evaluation framework nor the reviewing of the individual apps. In future iterations, the inclusion of patients would improve the quality of data collected. These limitations can inform future research to collect data on the users of these apps to draw more insights on how often the apps were used and the types of users and their likelihood to have better patient outcomes.

## Conclusions

Overall, there is a lack of evidence-based information and integration that enhance the patient-provider relationship in the CBT mobile app marketplace. Many apps only perform one function, mainly for patient engagement, and lack the functionality necessary to help patients adhere to their treatment within the larger health system. App developers should take note of the importance of evidence-based functionalities to improve patient outcomes which would encourage insurers and payers to begin to reimburse for the use of these technologies. Integration and connectivity with clinicians may facilitate improved app desirability and performance.

## Appendix 1

See Table 2.

**Table 2:** Apps downloaded for analysis

App name	Version code	Version date	App store download
ABC-Schema	2.2	31-Oct-18	Android
Aventurine Mood Improver	2.0.0	09-Aug-15	Android
Catch it	1.7	12-Oct-18	Apple
CBT Diary	2.0	27-Jul-18	Apple
CBT Journal	2.0	27-Jul-18	Apple
CBT Thought Record Diary	2.0	27-Jul-18	Apple
CBT-i Coach	2.3	14-Mar-18	Apple
Cogni	1.1.9	28-Jun-18	Apple
Cognitive Diary CBT Self-Help	4.2.5	07-Nov-16	Android
Cognitive Styles CBT Test	3.1	31-May-17	Android
Depression Self Help Guide: CBT	1.0	11-Dec-16	Android
End Anxiety Hypnosis	2.31	26-Sep-18	Android
Good Blocks	2.1	27-Oct-15	Apple
Happify	2.6.17	15-Oct-18	Apple
Happy Habits: Choose Happiness	2.2.1	12-May-14	Android
Jitters CBT	1.0.3	28-Oct-17	Apple
Joyable	3.5.2	01-Nov-18	Apple
Lantern	6.11.0	01-Aug-18	Apple
Merrier	2.2	24-Oct-18	Apple
Mindbliss	2.15.0	13-Oct-18	Apple
Moodpath: Depression	2.6.1	18-Oct-18	Apple
MoodTools—Depression Aid	1.4.2	26-Oct-18	Apple
NIH Depression Information	3.0.0.01	24-Mar-11	Android
Pacifica	7.0.7	22-Oct-18	Apple
See Betty	1.01	26-Jun-17	Apple
Self Esteem Blackboard	3.0	03-May-13	Android
STOPP app	0.0.3	20-Feb-18	Apple
Stress and Anxiety Companion	2.0	16-Oct-18	Apple
TF-CBT Triangle of Life	3.1	10-Nov-17	Apple
The Butterfly Diary	2.1.4	04-Dec-17	Apple
What’s Up?	2.3.1	05-Dec-16	Apple
Woebot	2.2.7	31-Oct-18	Apple
Wysa	4.1	29-Oct-18	Apple
Youper	6.05.004	24-Oct-18	Apple

## Abbreviations

CBT: cognitive behavioral therapy
iCBT: internet-based cognitive behavioral therapy
PREMs: patient reported experience measures
PROMs: patient reported outcome measures
